# Liensinine Ameliorating Oxidative Damage to Vascular Endothelium In Vitro via Upregulating eNOS and Nrf2/HO‐1 Signaling

**DOI:** 10.1002/fsn3.72013

**Published:** 2026-06-08

**Authors:** Feng Gao, Fengjiao Deng, Qiaowei Du, Tianya Xia, Yayi Deng, Shuang Liu, Bin Yu, Chenxiao Shan

**Affiliations:** ^1^ Department of Cardiology Wuxi Hospital Affiliated to Nanjing University of Chinese Medicine Wuxi China; ^2^ Jiangsu Key Laboratory of Efficacy and Safety Evaluation of Traditional Chinese Medicine Nanjing University of Chinese Medicine Nanjing China; ^3^ School of Pharmacy Nanjing University of Chinese Medicine Nanjing China

**Keywords:** eNOS, liensinine, network pharmacology, Nrf2/HO‐1 signaling, oxidative damage to vascular endothelium

## Abstract

Liensinine, as a main alkaloid from *Plumula nelumbinis*, shows a significant effect against vascular—related diseases, especially oxidative damage to vascular endothelium (ODVE). However, the underlying mechanism is unknown. The targets of liensinine against ODVE, the binding energies and molecular dynamics simulation between the proteins and liensinine were researched in the present study. The result was validated by protecting H_2_O_2_‐injured HUVECs via measuring cell viability, migration, apoptosis, contents of ROS and NO, expressions of Nrf2, HO‐1, SOD2, eNOS and p‐eNOS, and Nrf2 localization. SNP, L‐NAME, and ML385 were used as NO donor, eNOS inhibitor and Nrf2 inhibitor to validate the effect of liensinine in regulating Nrf2/HO‐1 pathway and eNOS activity. Venn analysis identified 77 common targets between liensinine and ODVE. According to the results of PPI analysis and molecular docking, liensinine possessed strong affinities to NFKB1, NFE2LE, and ESR1. Molecular dynamics simulation result indicated that NFE2LE–liensinine was the most stable complex. GO and KEGG enrichment results indicated that NO generation and oxidative stress were the main mechanisms involved in the protection. In the in vitro validation experiment, liensinine exhibited a strong protective effect against ODVE, including enhancing cellular activities, migration and NO generation, reducing ROS production and cellular apoptosis. The underlying mechanism might be associated with the upregulation of eNOS and Nrf2/HO‐1 pathway, which may even exhibit cross‐talk modulation. These findings reveal that liensinine has a strong potential to be used as a dietary supplement for patients with injured vascular endothelium due to oxidative stress.

## Graphical abstract

The present studies of network pharmacology, molecular docking and molecular dynamics simulation indicated that liensinine might ameliorate oxidative damage to vascular endothelium (ODVE) by regulating the targets of Nrf2, which is encoded by the *NFE2L2* gene. Then the in vitro study further showed that liensinine exhibited an obvious protection against H_2_O_2_‐induced ODVE, and the potential mechanism was related to the upregulation of Nrf2/HO‐1 and eNOS signaling. The results suggest that liensinine might be used as a dietary supplement for patients with injured vascular endothelium due to oxidative stress.

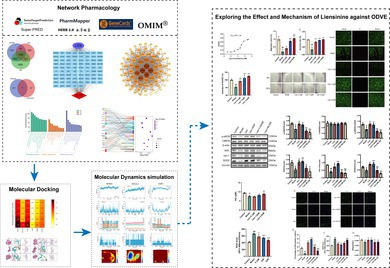

## 1 Introduction

Vascular endothelial cells are a crucial constituent part of the vascular system, located on the inner lining of blood vessels. Numerous studies have shown that they not only serve as a conduit system maintaining blood circulation, but also participate in regulating the physiological function by releasing active factors. Aging, inflammatory responses, abnormal glucose and lipid metabolism, and oxidative stress are all important causes of vascular endothelial dysfunction. Among these, oxidative stress plays a primary role (Zhou et al. 2023). If oxidative damage to vascular endothelium (ODVE) is not promptly reversed, it can further lead to vascular, especially microvascular, dysfunction, resulting in reduced NO production and alterations in the anti‐adhesion, anticoagulant, and vasodilatory functions of endothelial cells. This endothelial perturbation further induces reactive oxygen species (ROS) production, cellular antioxidant capacity impairment, membrane depolarization, ionic imbalance, cellular edema, cytoskeletal disruption, and inflammatory cell recruitment and activation, ultimately culminating in apoptotic cell death (Zhao et al. 2003; Baldea et al. 2018). Currently, the common vascular protective treatments in the clinic primarily include antiplatelet (Passacquale et al. 2022), hypolipidemic (Michaeli et al. 2023), antihypertensive (Zhang et al. 2026), and inflammation‐inhibiting medications (Welsh et al. 2017). However, the direct strategies to protect vascular endothelial cells from oxidative stress damage are still insufficient. Therefore, the identification of effective therapeutic agents to alleviate vascular oxidative stress‐induced damage is of considerable significance for preserving vascular function.

The lotus plumule, the seed embryo of *Nelumbo nucifera* Gaertn, is widely cultivated across regions. In daily practice, it is commonly utilized as a dietary ingredient or infused as a beverage, and it serves not only as a conventional food in East Asia but also as a frequently prescribed crude drug in traditional Chinese medicine. Its medicinal application as a tonic was first documented as early as the 16th century in the book named Ben‐Cao‐Gang‐Mu, particularly for its prominent antioxidant and inflammatory inhibition features (Chen, Fan, et al. 2019). Accumulating evidence has confirmed that the alkaloid components of lotus plumule exert diverse bioactivities, including alleviation of acute liver injury and mitigation of depressive emotion (Liu et al. 2019; Chen, Guo, et al. 2019). Liensinine was first isolated and structurally characterized by Chao et al. (1962). Subsequent studies have elucidated its broad‐spectrum pharmacological activities, such as attenuating oxidative stress (Wang, Yang, et al. 2023), protecting cardiomyocyte (Zhang et al. 2023; Shen et al. 2023), mitigating brain and liver injuries (Wang, Sun, et al. 2023; Zhang, Yuan, et al. 2024), and ameliorating hypertension‐induced vascular remodeling (Chen et al. 2025). However, whether liensinine can alleviate ODVE, suppress endothelial cell apoptosis, and restore endothelial function remains to be elucidated.

As a hypothesis‐generating tool, network pharmacology shifts research paradigms from a “mono‐target” to a “multi‐target” perspective (Li et al. 2011). It establishes a systematic discovery pipeline encompassing ingredient collection, target intersection, protein–protein interaction (PPI) analysis, and enrichment performance. This pipeline enables a bottom‐up exploration of the potential therapeutic mechanisms of Traditional Chinese Medicine (TCM) compounds. Notably, the predictive insights derived from network pharmacology serve as a foundational starting point, which requires subsequent experimental validation to confirm their biological relevance.

Accordingly, this study utilized a network pharmacology strategy to identify the key targets of liensinine in mitigating ODVE. Molecular docking and molecular dynamics simulation were then employed to evaluate the binding affinities and the stability between liensinine and the predicted potential proteins. Finally, the regulatory effects of liensinine against ODVE and its underlying molecular mechanisms were experimentally validated using an in vitro oxidative injury model of human umbilical vein endothelial cells (HUVECs).

## 2 Materials and Methods

### 2.1 Drugs and Reagents

HUVECs and the complete medium were obtained from Nanjing Saihongrui Biotechnology Co. Ltd. The medium consisted of 93% ECM (endothelial cell medium), 5% FBS (fetal bovine serum), 1% ECGS (endothelial cell growth supplement), and 1% P/S (penicillin/streptomycin). Endothelial cell basal medium (210–500) was obtained from Cell Applications Inc. H_2_O_2_ solution (H792072) and sodium nitroprusside (SNP, purity > 98%, S817931) were bought from Shanghai Macklin Biochemical Technology Co. Ltd. Liensinine (B20730), L‐NAME (S20013), and ML385 (S86700) were all purchased from Shanghai Yuanye Company. Liensinine powder was dissolved in DMSO to prepare a stock solution at 2 mg/mL (3.2747 mM). The stock solution was aliquoted and stored frozen. Before each experiment, it was thawed and diluted with basal medium to the indicated concentrations. The other drugs were prepared similarly according to their corresponding concentrations.

### 2.2 Screening Liensinine Targets

The targets of liensinine were collected via the four databases, including the SwissTargetPrediction (probability > 0.05), SuperPred (probability ≥ 0.6 and model accuracy ≥ 0.9), Pharmapper, and Herb 2.0 databases. Then these targets were merged, and the duplicates were removed.

### 2.3 Collecting the Targets of ODVE

The potential targets of ODVE were downloaded from the OMIM and Genecards databases using the keywords of “oxidative damage to vascular endothelium,” “oxidative stress,” and “vascular disease”, respectively. The genes were collected from the Genecards database using a cutoff of relevance score > 7.0 (Li et al. 2026; Ma et al. 2023). For each keyword, these collected targets from the two databases were combined, and the duplicates were removed. Then, the intersection targets of these three keywords were obtained by using Venn analysis and were defined as disease targets.

### 2.4 Venn Analysis and Constructing the Drug‐Target‐Disease and PPI Networks

Venn analysis was performed to obtain the overlapping targets between liensinine and disease. The “Drug‐Target‐Disease” network was presented by the Cytoscape software. Then, the PPI analysis was executed via the STRING platform (medium confidence). The plugins of CytoHubba and Centiscape 2.2 were employed for topology and degree analysis to obtain the crucial targets, which were visualized by the Cytoscape software.

### 2.5 Gene Ontology (GO) Functional and Kyoto Encyclopaedia of Genes and Genomes (KEGG) Pathways Enrichment Analyses

GO function terms and KEGG pathway were obtained via the Metascape website, in which GO analysis included three categories of biological process (BP), cellular component (CC), and molecular function (MF). The results were visualized via the Wei‐Sheng‐Xin platform (Tang et al. 2023).

### 2.6 Molecular Docking

The prepared files of the top 6 targets were obtained via the RCSB and TCMSP databases. The docking process was performed by the Autodock vina 1.2.3. It is a strong affinity if the binding energy < −7.0 kcal/mol (Ruan et al. 2024). PyMOL 2.3.2 and LigPlot 2.3.1 were employed to visualize the results.

### 2.7 Molecular Dynamics Simulation

In our molecular dynamics simulations, the PDB files of protein–ligand complexes that exhibited the most stable binding modes were utilized. The analysis was conducted using GROMACS 2023.2 and Python 3.11, where the AMBER99SB ILDN force field was applied to the proteins and the general Amber force field (GAFF) was used for the ligands. After generating the .gro, .itp, and .top files, the simulation box was solvated with water molecules along with Na^+^ and Cl^−^ ions. To ensure system stability, energy minimization was carried out over 5000 steps using the steepest descent algorithm. Following heating to 300 K, the NVT ensemble with the leap‐frog integrator was employed to maintain structural stability. The NPT ensemble kept the system under a pressure of 1 Pa and a temperature of 300 K. Both NVT and NPT equilibration phases lasted 100 ps each. Thereafter, each complex pair was subjected to a 100 ns molecular dynamics simulation. Subsequently, GROMACS was used to assess conformational evolution by calculating the root mean square deviation (RMSD), root mean square fluctuation (RMSF), radius of gyration (Rg), free energy landscape (FEL), and the number of hydrogen bonds. Finally, DuIvyTools was employed to generate the figures.

### 2.8 HUVECs Grouping, H_2_O_2_ ‐Induced Injury and Drug Treatment

#### 2.8.1 Research on Median Effective Concentration (EC_50_ ) Calculation

HUVECs were incubated with the medium containing different concentrations of liensinine (0.01, 0.1, 10, 1 × 10^2^, 5 × 10^2^, 1 × 10^3^, 5 × 10^3^, 5 × 10^4^, and 1 × 10^5^ nmol/L) for 24 h. Then, the cellular activity was detected, and the EC_50_ of liensinine was calculated using the Graphpad 8.3.0 software.

#### 2.8.2 Method of H_2_O_2_ ‐Induced HUVECs Injury

Cells in the logarithmic growth phase were seeded into the corresponding plates. After 24 h of attachment, the original complete medium was discarded, and the cells were washed twice with PBS. Then, cells were incubated with the complete medium containing a final concentration of 200 μM H_2_O_2_ and different concentrations of drugs for 24 h. After the 24 h exposure, subsequent measurements were performed. The control group received neither H_2_O_2_ nor the drugs, only an equal volume of complete medium.

#### 2.8.3 Research on Cellular Activity, Migration and Productions of ROS and Nitric Oxide (NO)

HUVECs were grouped into the control group, model group and liensinine groups (0.1, 1.0, and 5.0 μM). Specifically, (1) Control group: The cells were cultured in complete medium without H_2_O_2_‐injury and liensinine‐treatment. (2) Model group: The cells were cultured in complete medium containing 200 μM H_2_O_2_ for 24 h. (3) Liensinine groups: The cells were incubated with 200 μM H_2_O_2_ and liensinine for 24 h. The concentrations of liensinine were 0.1, 1.0, and 5.0 μM according to the corresponding groups.

#### 2.8.4 Research on Nuclear Factor Erythrocyte 2‐Associated Factor 2 (Nrf2)/heme Oxygenase‐1 (HO‐1) Signaling and Endothelial Nitric Oxide Synthase (eNOS) Regulations

HUVECs were allocated into six groups to validate the regulations on the Nrf2/HO‐1 and eNOS pathways, including the control, model, liensinine (5.0 μM), liensinine + SNP (100 μM), liensinine + L‐NAME (100 μM), and liensinine + ML385 (5.0 μM) groups, in which SNP, L‐NAME and ML385 were used as NO donor, e‐NOS inhibitor and Nrf2 inhibitor, respectively. The methods of H_2_O_2_‐induced cellular injury and treatment by drugs were similar to those mentioned above.

### 2.9 Cellular Activity and Migration Assays

Cellular activity was detected by a CCK‐8 kit (C0038, Beyotime, China). Specifically, after drug treatment and H_2_O_2_ injury, the cells were washed twice with PBS, and 10 μL of CCK‐8 solution was added to each well to achieve a final concentration of 750 μM for its core component of WST‐8 [2‐(2‐Methoxy‐4‐nitrophenyl)‐3‐(4‐nitrophenyl)‐5‐(2,4‐disulfophenyl)‐2H‐tetrazolium sodium salt]. After incubation for 2 h, the OD value was measured at 450 nm via a Synergy HT fluorescent microplate reader (BioTek Instruments, USA). Cell viability in each group was calculated relative to the control group, which was set as 100%.

HUVECs in the logarithmic growth phase were seeded into 24‐well plates at a density of 5 × 10^4^ cells per well. After 24 h, drug administration and H_2_O_2_ injury were performed as mentioned above. Subsequently, a sterile 200 μL pipette tip was used to create a scratch perpendicular to the bottom of the well. The cells were washed three times with PBS to remove detached cells, and the medium was replaced with serum‐free medium for further culture. At 0 and 12 h after scratching, the cells were washed three times with PBS, and the scratch width was observed under a microscope and photographed. The wound area was measured using ImageJ software. The migration area (%) = (scratching area at 0 h–scratching area at 12 h)/(scratching area at 0 h) × 100%. The percentage of migration area in the control group was set as 100%, and the migration area in other groups was calculated relative to the control.

### 2.10 Assessment of Apoptosis and Intracellular ROS

Cellular apoptosis was visualized using a calcein‐AM/propidium iodide (PI) kit (KTA1001, Abbkine, China). Following liensinine treatment and H_2_O_2_ injury, HUVECs were incubated with 200 μL per well of the working solution containing 0.1 μM calcein‐AM and 7.5 μM PI at 37°C in the dark for 20 min. Live cells and apoptotic cells were labeled with green and red fluorescence, respectively, under the fluorescence microscope (Nikon Ti‐S, Nikon, Japan).

The ROS contents were quantified with a ROS Kit (S0034S, Beyotime, China). Specifically, HUVECs were treated with either liensinine alone at different concentrations, or co‐treated with liensinine + SNP, liensinine + L‐NAME, or liensinine + ML385. After these treatments, cells were incubated with 10 μM of the working solution to detect the concentration of intracellular ROS using the fluorescent microplate reader.

### 2.11 Measurement of NO

Intracellular NO concentrations were quantified using a NO Kit (AKM005M, Boxbio, China). After co‐treatment with liensinine and H_2_O_2_, HUVECs were rinsed with PBS and subsequently lysed. The NO content was then determined by measuring the OD value at 550 nm.

### 2.12 Western Blotting Assay

After the total protein was collected and quantified, it was separated and transferred to the PVDF membrane. Then, the membrane was blocked and probed with primary antibodies against Nrf2 (1:500, 16396‐1‐AP, Proteintech, China), HO‐1 (1:500, 10701‐1‐AP, Proteintech, China), superoxide dismutase 2 (SOD2, 1:500, 24127‐1‐AP, Proteintech, China), eNOS (1:500, 345227, Zenbio, China), p‐eNOS (1:500, PA5‐104858, Invitrogen, USA), β‐actin (1:5000, AC026, Abclonal, China), and vinculin (1:500, CY5164, Abways, China). After incubation with the secondary antibody (1:10000, RGAR001, Proteintech, China), the blots were displayed via ECL chemiluminescence and SCG‐W2000 Chemiluminescence imaging instrument (Servicebio, China).

### 2.13 Nuclear Translocation of Nrf2

To assess the nuclear translocation of Nrf2, the cells were fixed, blocked, and incubated with the antibody against Nrf2 (1:400, 12721S, CST, USA) at 4°C. After they were probed with an Alexa fluor 488‐conjugated secondary antibody (1:500, ab150077, Abcam, UK) and counterstained with DAPI (C1006, Beyotime, China), the fluorescent images were captured.

### 2.14 Statistical Analysis

GraphPad Prism 8.0 was utilized for data analysis. Quantitative data are shown as mean ± SD. ANOVA and subsequent Tukey's post hoc test were employed to compare differences across multiple groups, with *p* < 0.05 set as the threshold for statistical significance.

## 3 Result

### 3.1 Targets Collection of Liensinine and ODVE

**FIGURE 1:**
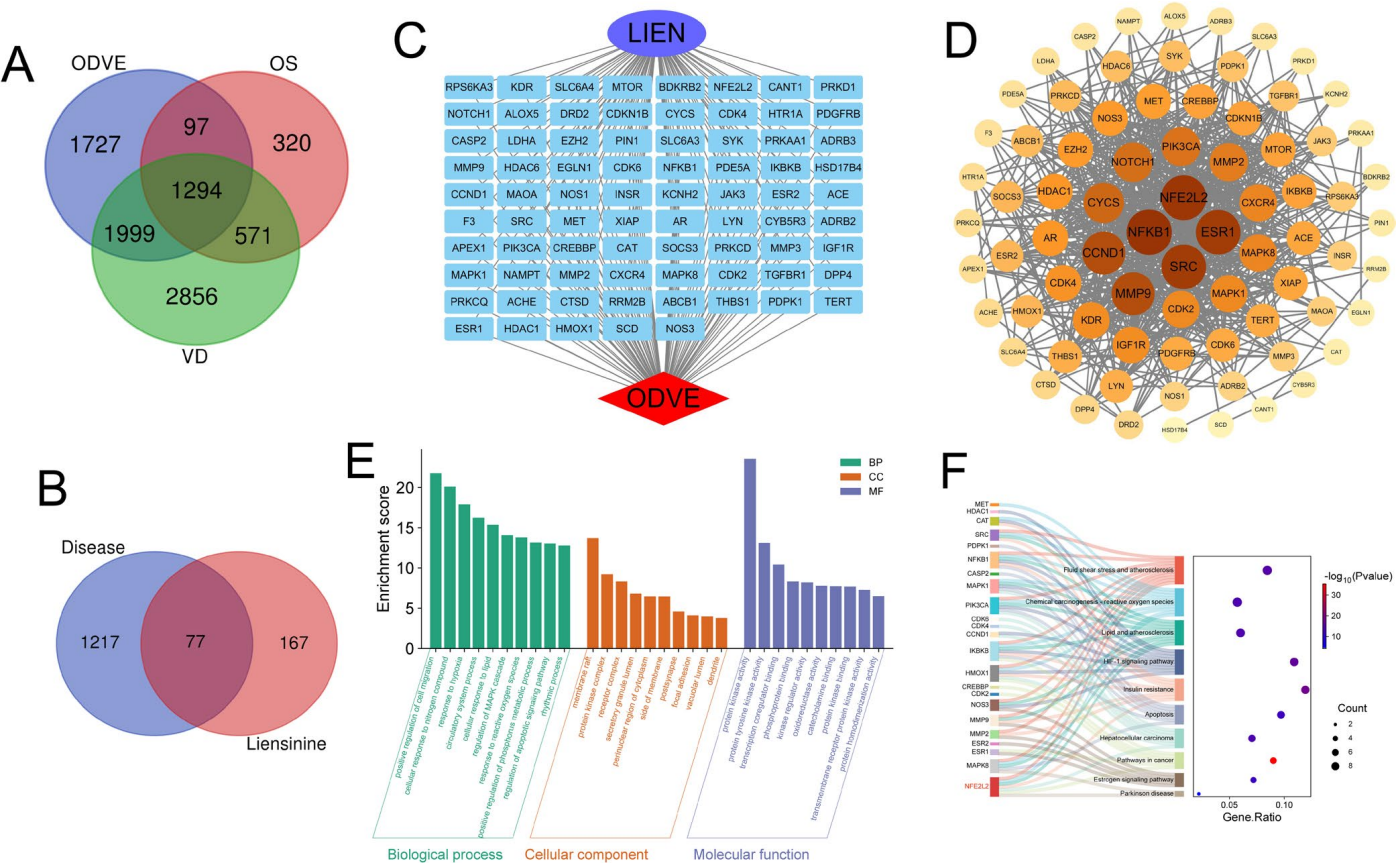
The results of network pharmacology analysis on liensinine against ODVE. (A) 1294 intersection targets were collected by Venn analysis among ODVE, OS and VD. (B) 72 common targets between liensinine and ODVE were screened by Venn analysis. (C) Network construction of liensinine‐targets‐ODVE. (D) PPI network of shared targets, in which higher degree values were indicated by darker colors and larger font sizes. (E) Results of GO functional enrichment analysis. (F) Sankey plot showcasing the key pathways and involved core genes in liensinine‐mediated protection against ODVE. The dot plot showed the ratio of the number of genes in a specific pathway to the total number of key genes. ODVE, oxidative damage to vascular endothelium; OS, oxidative stress; VD, vascular disease.

As shown in Figure 1A, 1294 disease targets were screened via the Genecards and Omim databases. A total of 244 targets of liensinine were collected via the databases of SwissTargetPrediction, SuperPred, Pharmapper, and Herb 2.0. Subsequently, the Venn analysis result showed that there were 77 common targets between the compound and the disease, as shown in Figure 1B.

### 3.2 Constructing Networks of “Drug‐Targets‐Disease” and PPI

The network of “Drug‐Targets‐Disease” was constructed via Cytoscape software. As shown in Figure 1C, the liensinine was connected with ODVE via the 77 common genes, which suggested the mechanism of the drug against the disease was related to multi‐target regulation. In order to further analyze the relationship of the common targets, the PPI network was drawn using the STRING platform and visualized by Cytoscape software, as shown in Figure 1D. Meanwhile, node degree analysis was performed by the plugins of CytoHubba and Centiscape 2.2. Then, the top 6 key targets were obtained, including NFKB1, NFE2L2, ESR1, SRC, MMP9, and CCND1.

### 3.3 GO Function Enrichment and KEGG Pathway Enrichment

In order to further identify the potential biological function and pathway involved in the protection of liensinine against ODVE, the enrichments of GO and KEGG were performed. The results were displayed in Figure 1E. GO annotations included three classifications, including BP, CC, and MF. The key BP terms related to ODVE were cellular response to NO, response to hypoxia, circulatory system process, response to reactive oxygen species, and regulation of apoptotic signaling pathway, respectively. The key CC terms included membrane raft, protein kinase complex, receptor complex, and secretory granule lumen, respectively. The key MF terms included protein kinase activity, protein tyrosine kinase activity, transcription coregulator binding, and oxidoreductase activity, respectively. Then, the Sankey plot exhibited not only the results of KEGG but also the connection between the key pathways and the related core genes, as shown in Figure 1F. The Gene Ratio was between 0.02 and 0.12, which indicated that the core genes were enriched in the top 10 pathways. Specifically, NFE2L2, HMOX1, NFKB1, IKBKB, PIK3CA, NOS3, and MAPK8 were the genes which are involved in regulating the fluid shear stress and the atherosclerosis pathway, which was the top 1 pathway. Moreover, as the top 2 and top 3 pathways in KEGG enrichment, the chemical carcinogenesis‐reactive oxygen species pathway and the atherosclerosis pathway contained most of the above genes and MAPK1, CAT, MET, PIK3CA, and MAPK1. It is worth noting that although NFE2L2 ranks second in the PPI analysis, it is associated with the largest number of pathways in the KEGG analysis.

### 3.4 Molecular Docking

**FIGURE 2:**
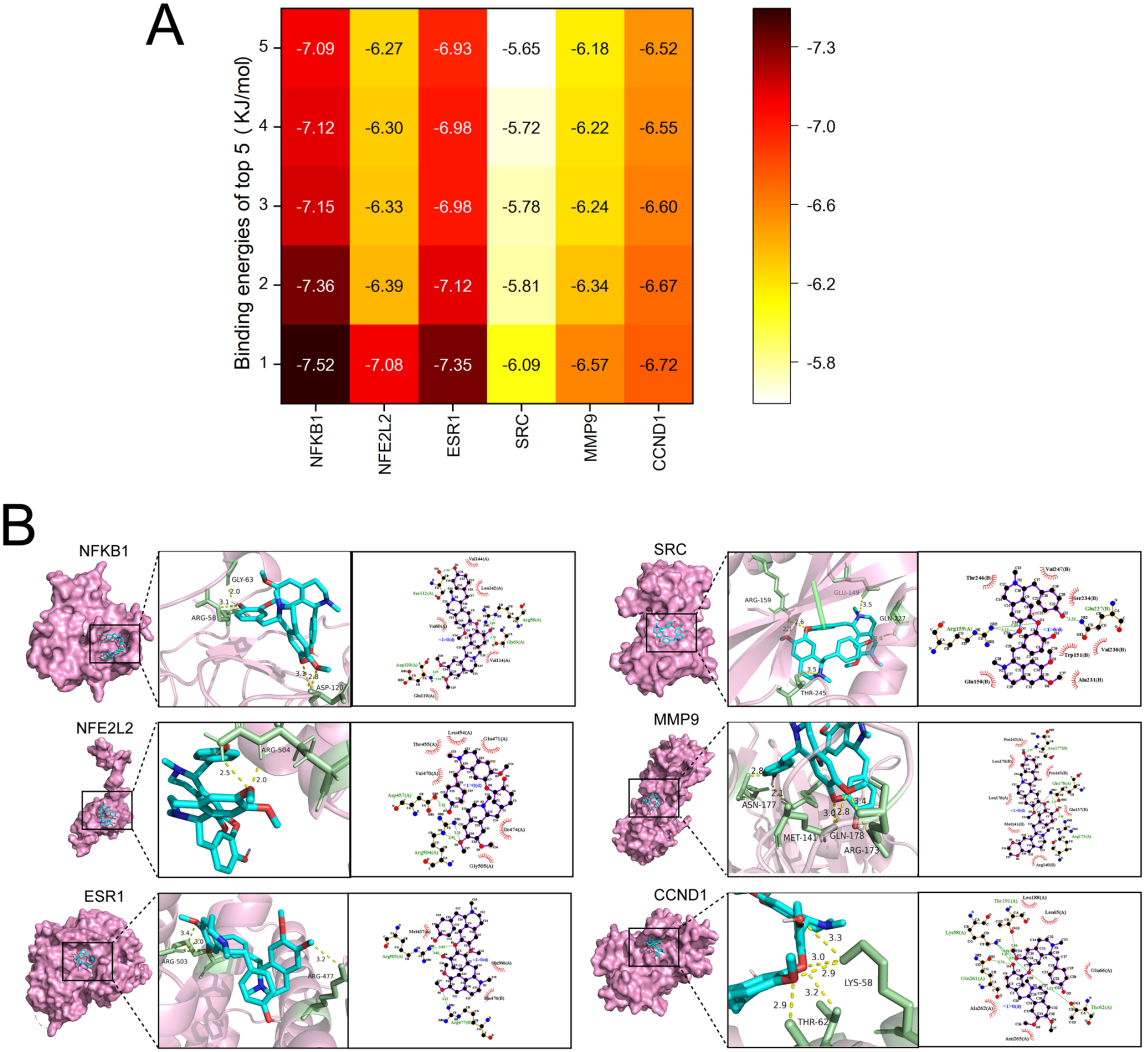
Molecular docking results. (A) The heatmap of the top 5 binding energies of each combination between liensinine and the 6 proteins of NFKB1, NFE2L2, ESR1, SRC, MMP9, and CCND1, respectively. (B) The present binding modes of each combination between liensinine and the proteins.

Molecular docking analysis was performed to detect the binding abilities between liensinine and the top 6 targets in PPI analysis, including NFKB1, NFE2L2, ESR1, SRC, MMP9, and CCND1, and the top 5 binding energies in each pair were exhibited in Figure 2A. The binding energy between a ligand and a protein less than −5.0 kcal/mol is considered good affinity, while less than −7.0 kcal/mol is considered to be a strong affinity. The result showed that the drug exhibited good affinity to all of the six proteins, in which its binding to NFKB1, NFE2L2, and ESR1 exhibited strong affinity, as shown in Table 1. The visualizations of the docking mode of each protein with the strongest affinity were shown in Figure 2B.

**TABLE 1:** Binding energies between liensinine and the potential targets.

Targets	Top 5 binding energies (KJ/mol)				
	Top 1	Top 2	Top 3	Top 4	Top 5
NFKB1	−7.52	−7.36	−7.15	−7.12	−7.09
NFE2L2	−7.08	−6.39	−6.33	−6.30	−6.27
ESR1	−7.35	−7.12	−6.98	−6.98	−6.93
SRC	−6.09	−5.81	−5.78	−5.72	−5.65
MMP9	−6.57	−6.34	−6.24	−6.22	−6.18
CCND1	−6.72	−6.67	−6.60	−6.55	−6.52

### 3.5 Molecular Dynamics Simulation

**FIGURE 3:**
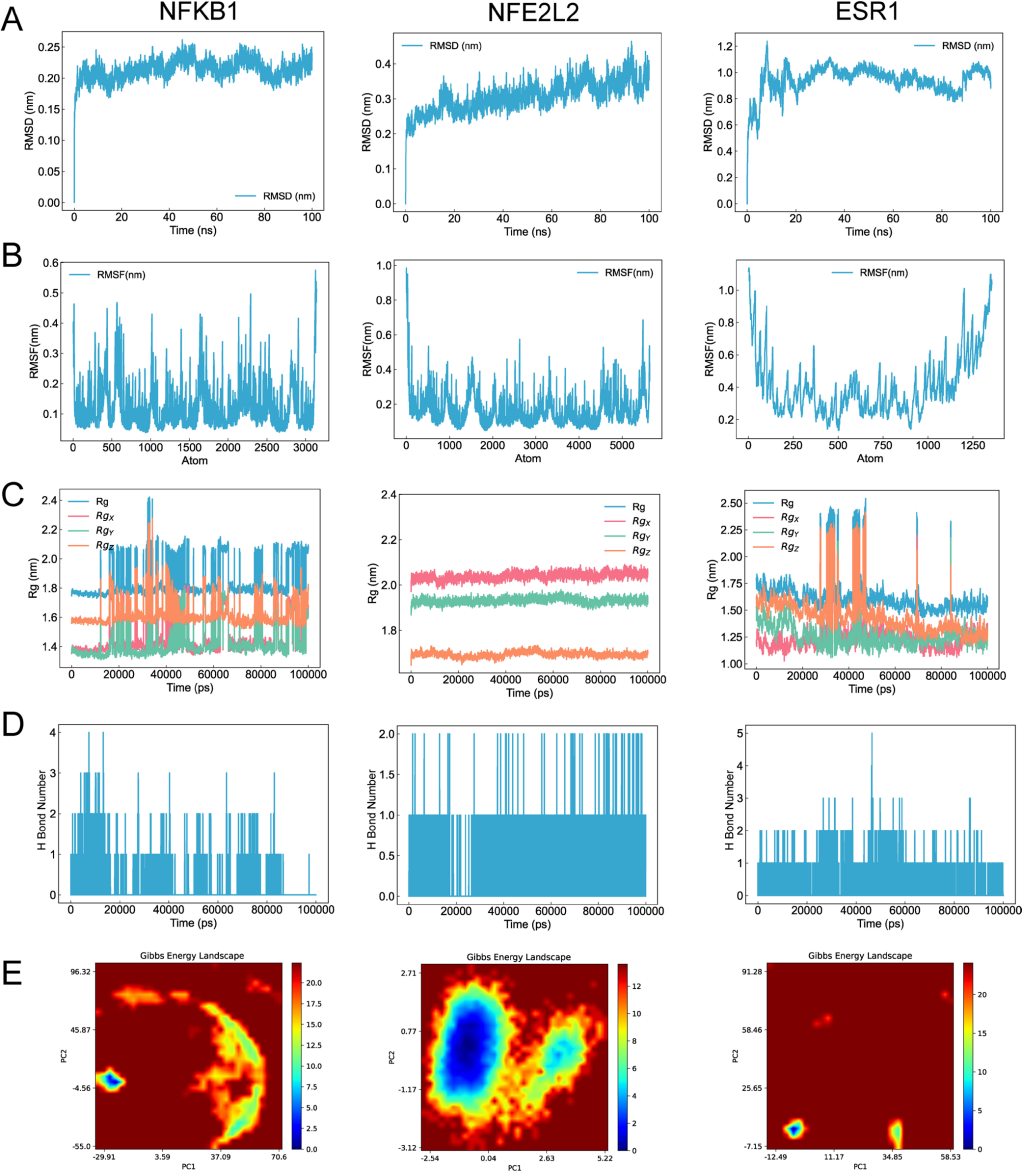
The results of molecular dynamics simulation between liensinine and NFKB1, NFE2L2, and ESR1 during 100 ns. (A) RMSD, (B) RMSF, (C) Rg, (D) hydrogen bond number, and (E) FEL were shown for each complex.

To evaluate the binding stability of the complex of protein and ligand, a 100 ns MDS was executed, and the five dynamic indices were calculated, including RMSD, RMSF, Rg, number of hydrogen bonds, and FEL, as displayed in Figure 3A–E. The RMSD of the complexes of NFKB1–liensinine and NFE2L2–liensinine reached equilibrium within 5 ns and maintained equilibration. Then, it exhibited only small fluctuations within 2 Å. While the complex of ESR1–liensinine showed a large fluctuation of more than 6 Å during the whole simulation. Apparently, the complexes of NFKB1–liensinine and NFE2L2–liensinine were much more stable than the ESR1–liensinine complex. The RMSF of the NFE2L2–liensinine complex remained very stable with the fluctuation less than 2 Å, while both the complexes of NFKB1–liensinine and ESR1–liensinine exhibited large fluctuation over 4 Å, which indicated that the former possessed lower flexibility and more structural rigidity than the latter two complexes. Similarly, the Rg of the NFE2L2–liensinine complex was more stable than those of the NFKB1–liensinine and ESR1–liensinine complexes, which displayed the obvious fluctuation of more than 6 Å. So, the overall structure of the NFE2L2–liensinine complex was much more stable than those of the other two complexes. Hydrogen bonds constitute one of the key stabilizing forces in protein–ligand binding. The number and duration of hydrogen bonds formed between the receptor and ligand during the simulation can be used to evaluate the stability of the interaction. Although the number of hydrogen bonds increased up to 4 for the NFKB1 complex, its duration was obviously shorter than those of the other two complexes, which were similar overall. PC1 and PC2 were used to map the energy minima in FEL analysis. The results showed that the NFE2L2 complex generated a conformational region with marked low free energy, while no structural perturbation was experienced in the other two complexes. Together, the above results indicated that the NFE2L2 complex exhibited a more stable conformation and hydrogen bond binding between the protein and liensinine than the NFKB1 and ESR1 complexes during the whole simulation period. So, the NFE2L2/HO‐1 pathway would be measured in the following mechanism research.

### 3.6 Results of EC_50_ Calculation and Cellular Activity Assays

**FIGURE 4:**
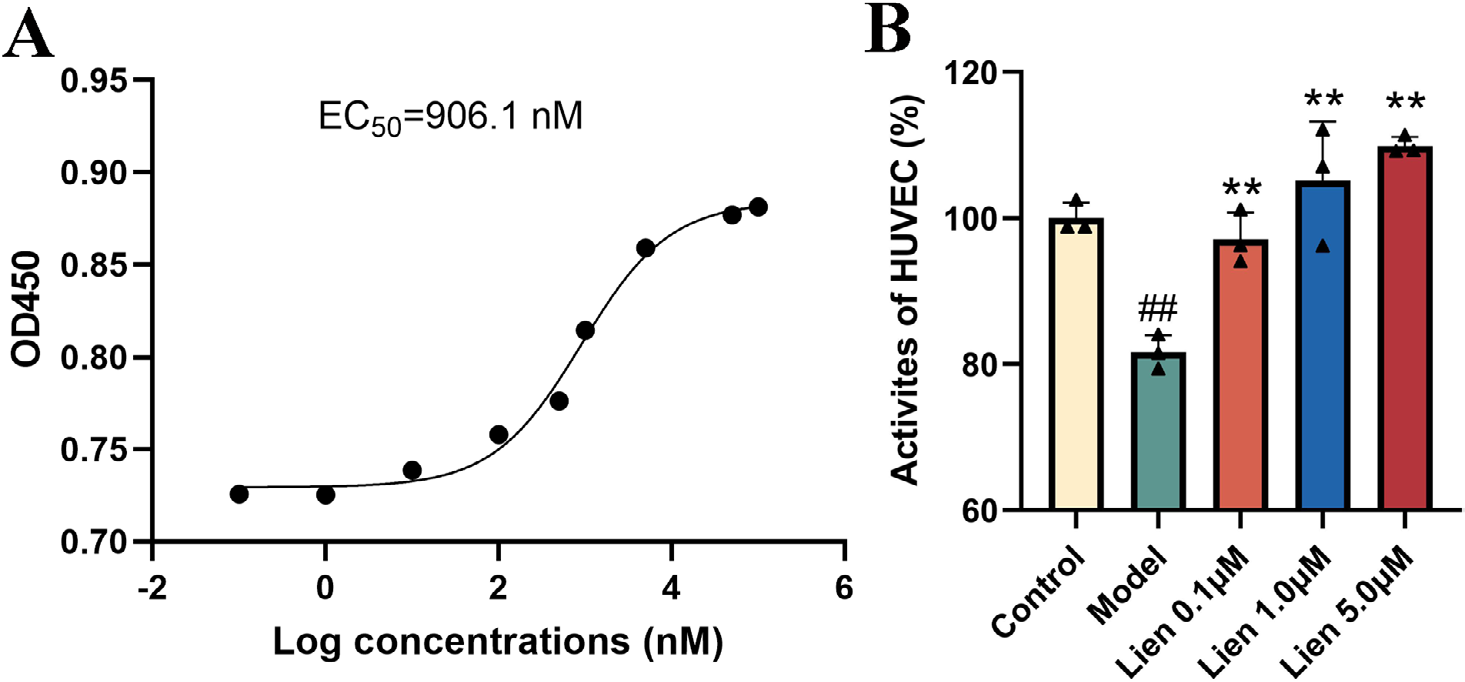
EC_50_ of liensinine and its effect in protecting H_2_O_2_‐injured HUVECs. (A) Calculation result of EC_50_ on liensinine. (B) Liensinine ameliorated the injury of H_2_O_2_ on the cellular activity (mean ± SD, *n* = 3). ^##^ *p* < 0.01 versus the control group; *p < 0.05, **p < 0.01 versus the model group. Lien, liensinine.

We first established the dose–effect curve of liensinine on normal HUVECs activity and obtained its EC_50_ of 906.1 nM, as shown in Figure 4A. Based on the EC_50_ result, 0.1, 1, and 5 μM of liensinine were selected to evaluate its improvement on the activity of H_2_O_2_‐injured HUVECs. The results showed that exposure to H_2_O_2_ significantly reduced cellular activity compared to controls (*p* < 0.01). However, liensinine treatment elicited a dose‐dependent recovery of cellular activity (Figure 4B).

### 3.7 Liensinine Enhanced HUVECs Migration and Survival Rate

**FIGURE 5:**
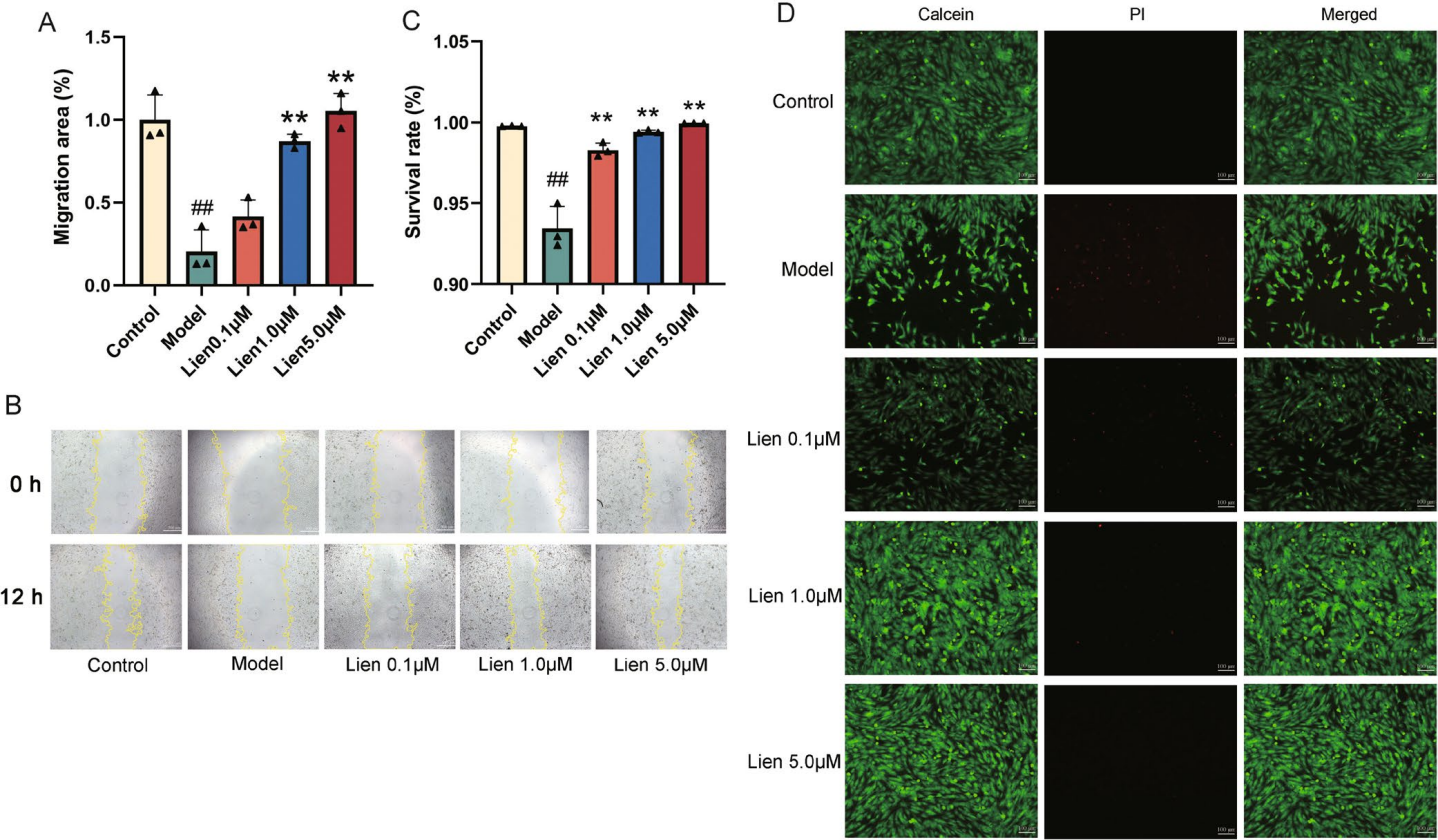
The migration promotion and apoptosis inhibition effect of liensinine. (A) The ratio of the scratch area of each group in 0 and 12 h after the cells were scratched (mean ± SD, *n* = 3). (B) The representative images of the scratching repair assay. (C) The survival ratio of each group to the control group (mean ± SD, *n* = 3). (D) The typical fluorescent images of the calcein‐AM/PI staining. ^##^ *p* < 0.01 versus the control group; *p < 0.05, **p < 0.01 versus the model group. Lien, liensinine.

To further evaluate the repair capability of liensinine against ODVE, we performed wound healing and apoptosis assays. The model group exhibited impaired migration ability in comparison to the control group (*p* < 0.01), while 1.0 and 5.0 μM of liensinine markedly increased the migration area (*p* < 0.01), as shown in Figure 5A,B. Moreover, H_2_O_2_ also reduced the survival ratio of HUVECs obviously (*p* < 0.01), and all of the three concentrations of liensinine elevated the cellular survival rate markedly (*p* < 0.01), characterized by increased cell density and restored morphology, as shown in Figure 5C,D.

### 3.8 Liensinine Mitigated Intracellular ROS Accumulation and Promoted NO Generation

**FIGURE 6:**
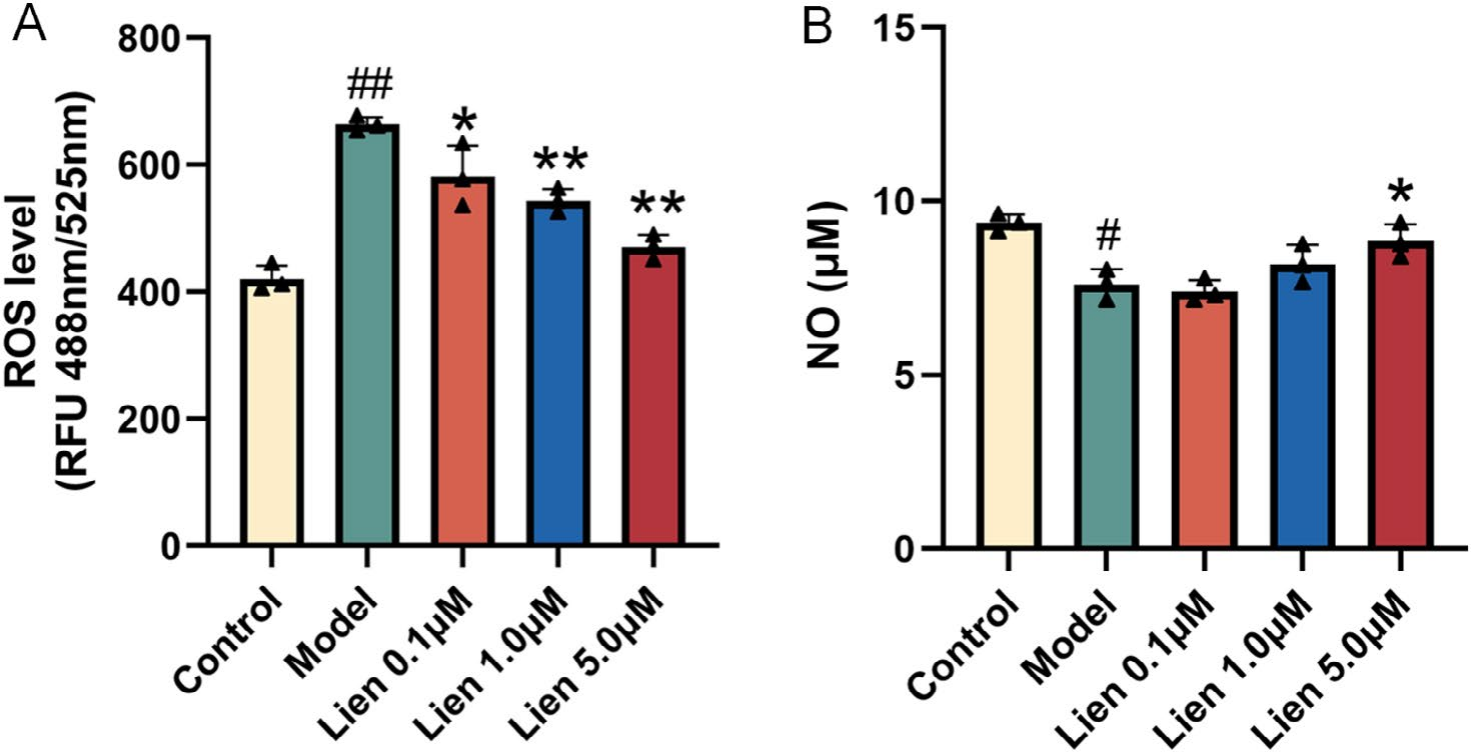
Liensinine reduced the intracellular ROS levels and enhanced the NO concentration (mean ± SD, *n* = 3). (A) The result of the intracellular ROS level in each group. (B) The result of the intracellular NO concentration in each group. ^#^ *p* < 0.05, ^##^ *p* < 0.01 versus the control group; *p < 0.05, **p < 0.01 versus the model group. Lien, liensinine.

Oxidative stress disrupts endothelial homeostasis by elevating ROS and depleting NO. Figure 6A,B showed an obvious increase in intracellular ROS levels and a reduction of NO concentration following H_2_O_2_ exposure (*p* < 0.05, 0.01). Three concentrations of liensinine significantly dose‐dependently suppressed ROS fluorescence intensity (*p* < 0.05, 0.01). Additionally, the high concentration of liensinine restored NO production compared to the model group (*p* < 0.05).

### 3.9 Liensinine Activated the Nrf2/HO‐1 Axis and Promoted eNOS Phosphorylation

**FIGURE 7:**
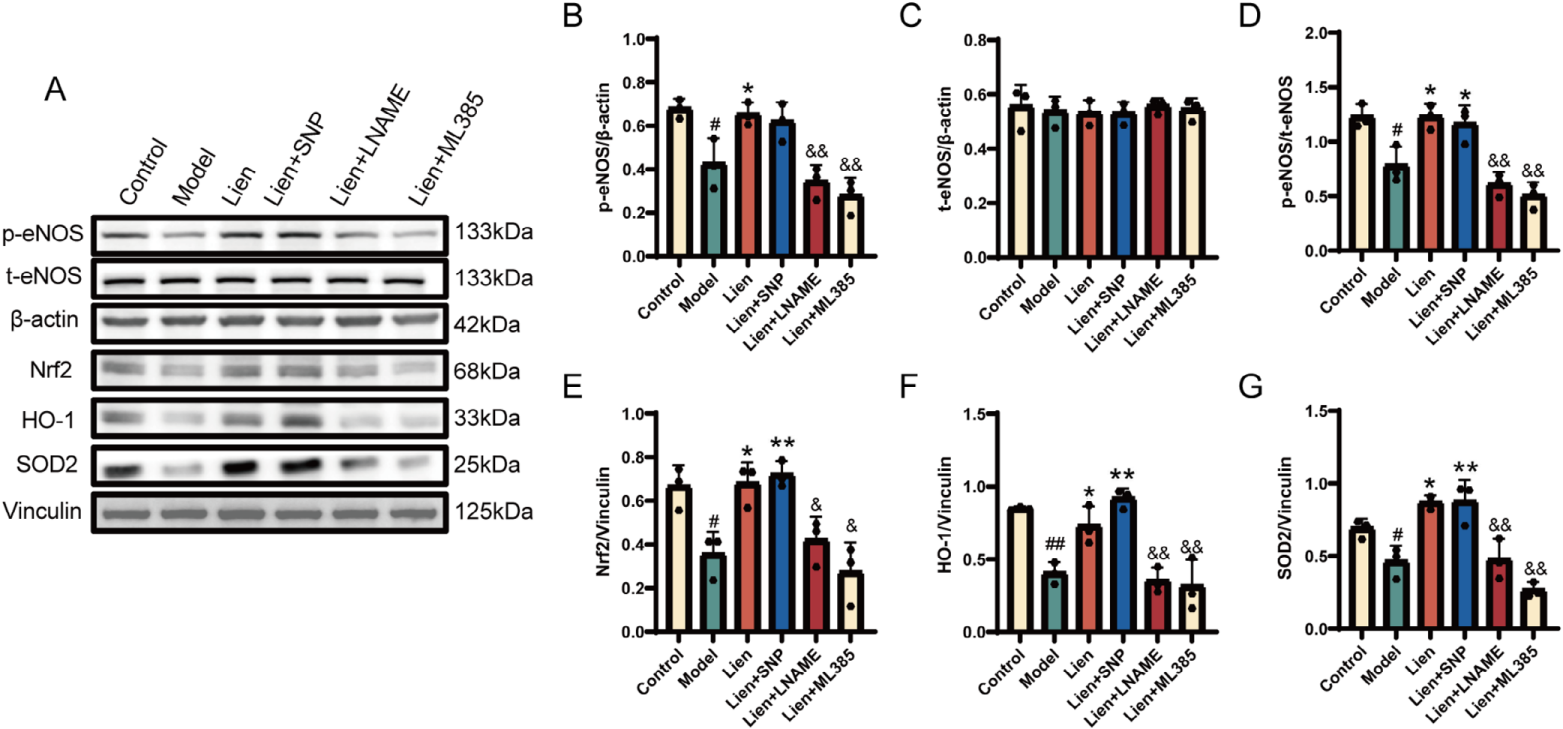
Liensinine activated the Nrf2/HO‐1 pathway and promoted eNOS phosphorylation. (A) The representative bands of p‐eNOS, t‐eNOS, Nrf2, HO‐1, SOD2, β‐Actin and vinculin in the western blots assay. (B–G) The relative expressions of p‐eNOS, t‐eNOS, Nrf2, HO‐1 and SOD2, and the ratio of p‐eNOS, t‐eNOS (mean ± SD, *n* = 3). ^#^ *p* < 0.05, ^##^ *p* < 0.01 versus the control group; *p < 0.05, **p < 0.01 versus the model group; ^&^ *p* < 0.05, ^&&^ *p* < 0.01 versus the liensinine group. Lien, liensinine; LNAME, L‐NAME.

The Nrf2/HO‐1 pathway is a crucial component of the antioxidant system. The results showed that H_2_O_2_ suppressed the expression of Nrf2, HO‐1, SOD2, p‐eNOS, and p‐eNOS/t‐eNOS in comparison to the control group (*p* < 0.05, 0.01, Figure 7A–G). Liensinine (5.0 μM) treatment significantly upregulated these proteins (*p* < 0.05), which suggested the upregulation effects of liensinine on Nrf2/HO‐1 signaling and eNOS phosphorylation. Interestingly, L‐NAME, as an inhibitor of eNOS, not only reduced p‐eNOS level, but also decreased Nrf2, HO‐1, and SOD2 expressions (*p* < 0.05, 0.01). Similarly, ML385, as an inhibitor of Nrf2, not only downregulated the Nrf2 and HO‐1 levels, but also lessened the eNOS phosphorylation and SOD2 level (*p* < 0.05, 0.01). Although the liensinine alone group enhanced the expressions of Nrf2 and HO‐1 compared to the model group (*p* < 0.05), the combination of liensinine and SNP treatment exhibited more obvious enhancement of the expressions of both proteins (*p* < 0.01), which indicated that there might be cross‐talk between the pathways of Nrf2/HO‐1 and eNOS.

### 3.10 Liensinine Promoted the Nuclear Translocation of Nrf2

**FIGURE 8:**
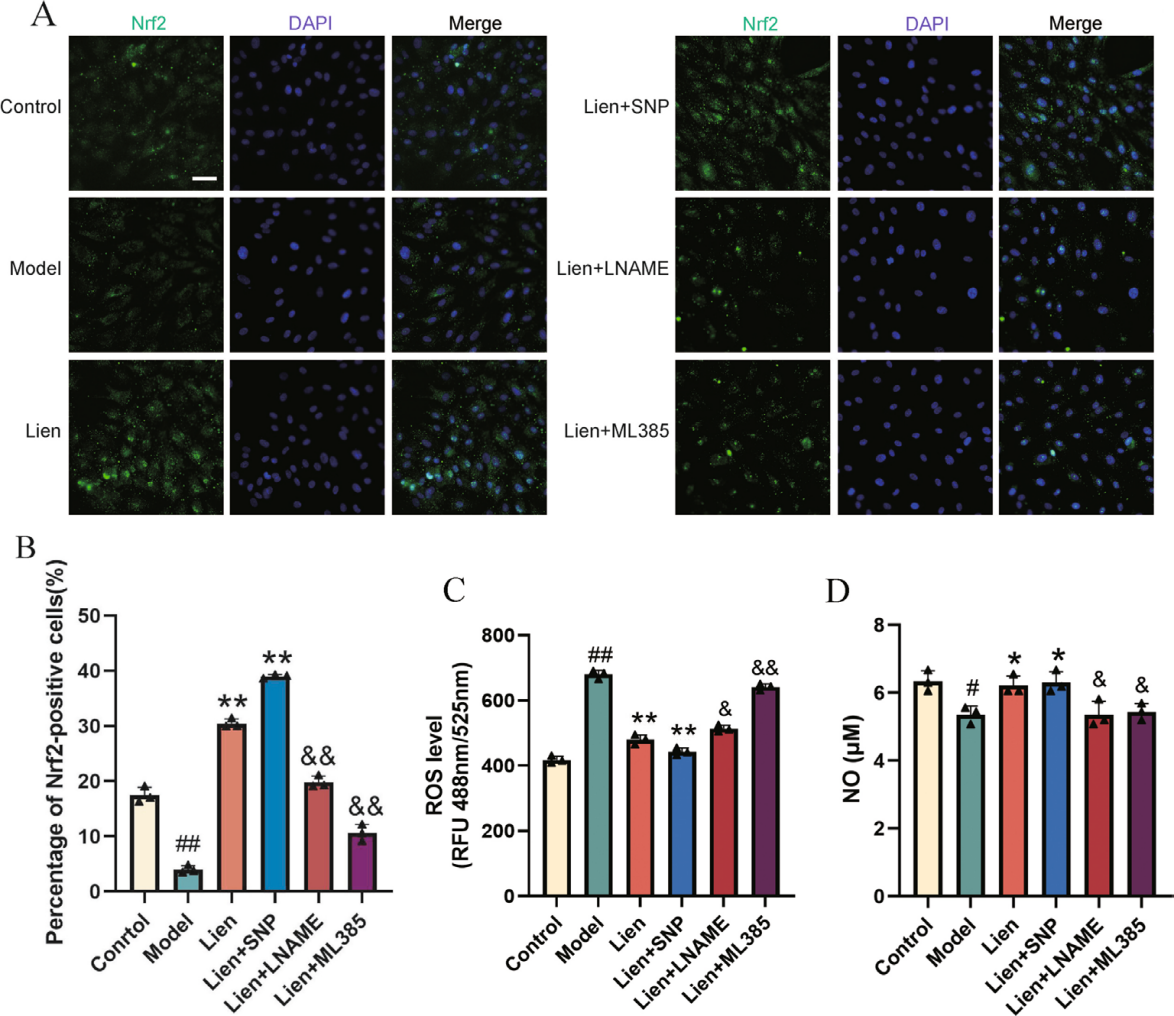
Liensinine promoted the nuclear translocation of Nrf2. (A) The representative immunofluorescence images of Nrf2/DAPI of HUVECs. (B) The result of the percentage of Nrf2/DAPI double‐positive cells (mean ± SD, *n* = 3). (C) The result of the intracellular ROS level in each group (mean ± SD, *n* = 3). (D) The result of the intracellular NO concentration in each group (mean ± SD, *n* = 3). ^#^ *p* < 0.05, ^##^ *p* < 0.01 versus the control group; *p < 0.05, **p < 0.01 versus the model group; ^&^ *p* < 0.05, ^&&^ *p* < 0.01 versus the liensinine group. Lien, liensinine; LNAME, L‐NAME.

An immunofluorescence assay was employed to observe the nuclear translocation of Nrf2, and Figure 8A,B detailed the results. Compared to the control group, Nrf2 was located predominantly in the cytoplasm in the model group (*p* < 0.01), while 5.0 μM of liensinine induced significant Nrf2 nuclear translocation (*p* < 0.01). This trend of translocation was potentiated by SNP intervention, but it was offset by L‐NAME and ML385 intervention. The results showed that NO deficiency might limit Nrf2 nuclear translocation, which confirmed the potential cross‐talk relationship between Nrf2/HO‐1 signaling and eNOS activation.

### 3.11 Crosstalk Between Nrf2\HO‐1 Signaling and eNOS Activation Involved in Liensinine‐Induced ROS Accumulation and NO Generation

It is well known that the reduction of intracellular ROS level could characterize the activation of Nrf2\HO‐1 signaling, while intracellular NO content could feature the activation of eNOS. To further verify the crosstalk between the downstreams of Nrf2\HO‐1 signaling and eNOS activation, and the regulation of liensinine on it, the ROS level and NO concentration were measured in the presence of SNP, L‐NAME or ML385. As depicted in Figure 8C,D, the combination of liensinine and SNP still showed a better trend in alleviating ROS accumulation, although both groups increased NO content and decreased ROS level (*p* < 0.05, 0.01). When treated with ML385, a well‐characterized Nrf2 inhibitor, the ROS‐reducing effect exerted by liensinine was effectively counteracted, as expected (*p* < 0.01, vs. liensinine group). Similarly, as an eNOS inhibitor, L‐NAME counteracted the NO‐elevating effect of liensinine, as expected (*p* < 0.05, vs. liensinine group). Interestingly, L‐NAME also offset liensinine‐induced ROS elimination (*p* < 0.05, vs. liensinine group), and ML385 offset liensinine‐induced NO creation (*p* < 0.05, vs. liensinine group). Apparently, the results further suggested that there might be cross‐talk between the Nrf2/HO‐1 signaling and eNOS activation, which was the pharmacological basis of liensinine treating OVDA.

## 4 Discussion

Over the past few years, a growing body of evidence has indicated that both physiological aging and numerous pathological processes are associated with oxidative stress responses in vascular endothelial cells, including hypertension (Uruski et al. 2025), hyperlipidemia (Cheng et al. 2023), hyperglycemia (Bai et al. 2025), inflammation (Abrashev et al. 2025), preeclampsia (Cao et al. 2025) and ischemia–reperfusion injury (Zhong et al. 2025). ROS are chemically reactive derivatives of molecular oxygen. At low levels under normal physiological conditions, these molecules are mainly generated as byproducts of routine cellular metabolism and serve dual beneficial roles. They aid in pathogen destruction and immune defense, while also functioning as secondary messengers that regulate intracellular signaling across diverse biological processes (Thannickal and Fanburg 2000). However, when the body's antioxidant defenses are compromised or when ROS are overproduced to an extent that overwhelms the neutralizing capacity of the antioxidant system, a state known as oxidative stress arises. Within endothelial cells, NO plays a central role in preserving vascular health. A decline in NO bioavailability, resulting either from diminished NO synthesis or from enhanced scavenging of NO by superoxide anions, triggers the initiation of endothelial dysfunction. ROS can originate from numerous sites inside the cell. The superoxide anion radical (O_2•_^−^) is the initial radical species formed, and it serves as the primary precursor for several other biologically significant oxidants within the vascular endothelium, such as H_2_O_2_, the hydroxyl radical (•OH) and peroxynitrite (ONOO^−^). Production of superoxide occurs when molecular oxygen undergoes partial reduction, which is a reaction catalyzed by multiple enzymatic systems, including the mitochondrial electron transport chain, NADPH oxidase, dysfunctional endothelial nitric oxide synthase (eNOS), and xanthine oxidase (Incalza et al. 2018). Evidently, the identification of pharmaceuticals or functional foods that can retard or mitigate these physiological and pathological oxidative processes holds considerable significance. Given the requirement for long‐term interventions against oxidative injury in vascular endothelial cells, it is of greater importance to explore bioactive components from functional foods as potential therapeutic agents.

Liensinine was first isolated from *Plumula nelumbinis*, which is documented in the Pharmacopeia of the People's Republic of China for its “clearing heart‐fire” effect in terms of the Traditional Chinese Medicine Theory, and is widely used as a functional food across Asia. Its chemical composition encompasses alkaloids, chlorophyll, flavonoids, organic acids, sterols, volatile oils, and trace elements (Meng et al. 2022). The total alkaloids in *Plumula nelumbinis* are classified into two categories: phenolic alkaloids and non‐phenolic alkaloids, with liensinine being a phenolic alkaloid of relatively high abundance. Previous studies have demonstrated that liensinine possesses a broad spectrum of biological activities, including blood pressure reduction (Chen et al. 1962), improvement of vascular remodeling (Jia et al. 2023), alleviation of type 2 diabetes mellitus (Luo et al. 2025), attenuation of Alzheimer's disease (Meng et al. 2022), and mitigation of inflammatory responses (Wang et al. 2024). From a chemical structural perspective, liensinine is the demethylated derivative of neferine. Specifically, liensinine contains one phenolic hydroxyl group and two methoxy groups, and methylation of its phenolic hydroxyl group yields neferine. Extensive studies have confirmed that neferine exerts a significant protective effect against oxidative stress‐induced injury in vascular endothelial cells (Tang et al. 2019; Guan et al. 2014). However, whether liensinine, owing to its extra phenolic hydroxyl group, exerts comparable antioxidant effects on vascular endothelial cells remains unreported to date.

Network pharmacology is implemented by integrating biological regulatory networks with drug‐target networks and analyzing the interactions between drugs and network nodes. This approach shifts the research paradigm from the pursuit of individual targets to comprehensive network analysis. By constructing network models, network pharmacology facilitates the exploration of active components in natural products, the prediction of their potential therapeutic targets, and the investigation of their disease correlations, thereby providing novel insights for drug development (Ruan et al. 2024). To date, no network pharmacology research on liensinine has been reported, let alone studies focusing on its protective effects against ODVE. In the present study, we collected 244 targets of liensinine and screened 1294 targets associated with ODVE, identifying 77 overlapping targets between the two sets. Following PPI analysis, the top 6 core targets were identified as NFKB1, NFE2L2, ESR1, SRC, MMP9, and CCND1. GO and KEGG enrichment analyses revealed that the chemical carcinogenesis‐reactive oxygen species pathway, fluid shear stress, and atherosclerosis pathway were the key mechanisms underlying liensinine‐mediated protection against ODVE. The key genes included NFE2L2, HMOX1, and NOS3, which encode Nrf2, HO‐1, and eNOS, respectively.

Although network pharmacology provides insights into the potential targets and active components mediating a drug's therapeutic effects, the binding stability between drugs and targets remains elusive. Molecular docking is widely recognized as a robust method for evaluating ligand‐protein binding affinity (Wang et al. 2025). Herein, we performed molecular docking analysis to assess the binding capacity of liensinine to the aforementioned 6 core targets. The results demonstrated that liensinine exhibited favorable binding affinity to all 6 proteins. Notably, its binding to NFKB1, NFE2L2, and ESR1 showed relatively strong affinity (binding energy < −7.0 kcal/mol). To further evaluate the stability of the three pairs of the complex, a 100 ns MDS was performed. The results suggested that the NFE2L2 complex exhibited a more stable conformation and hydrogen bond binding than the NFKB1 and ESR1 complexes during the whole period of simulation. So, the NFE2L2/HO‐1 pathway might be the key mechanism of liensinine against ODVE. Nevertheless, these *in silico* observations require further verification through in vitro experiments.

EC_50_ serves as a critical reference for evaluating drug safety and efficacy, as well as a fundamental basis for dosage selection. In the present study, the EC_50_ value of liensinine for promoting HUVEC proliferation was determined to be 0.906 μM. Therefore, concentrations of 0.1, 1.0, and 5.0 μM were selected for liensinine treatment in subsequent experiments. Under oxidative stress conditions, substantial amounts of hydrogen peroxide (H_2_O_2_) are generated. Excessive H_2_O_2_ accumulation induces vascular endothelial damage and triggers cellular apoptosis. Accordingly, exogenous H_2_O_2_ treatment is currently the most commonly used method to establish oxidative stress models in vascular endothelial cells (Feng et al. 2025). Our results demonstrated that H_2_O_2_ exposure significantly reduced HUVECs activity, whereas all three doses of liensinine markedly ameliorated this damage, confirming the compound's potent protective effects against ODVE.

The wound‐healing assay is a well‐established in vitro technique for investigating directional cell migration, and it is widely utilized by researchers due to its ability to simulate in vivo cell migration during wound repair (Gao et al. 2024). Calcein‐AM is a non‐fluorescent membrane‐permeable dye that can enter viable cells, where it is hydrolyzed by intracellular esterases to produce calcein, a metabolite with strong green fluorescence, thus enabling specific labeling of living cells. In contrast, PI is a nuclear‐staining dye that cannot penetrate intact cell membranes. However, it can cross the disrupted, permeable cell membranes of dead cells, enter the nucleus, intercalate into the DNA double helix, and emit red fluorescence. Hence, PI is used to label dead cells, especially late apoptotic cells (He et al. 2025). The present study confirmed that liensinine significantly enhanced HUVEC migration and suppressed cellular apoptosis, indicating that the drug's protective effects against ODVE are associated with anti‐apoptotic activity. Although Qiao reported that liensinine could reduce apoptosis in human cortical neurons induced by oxygen–glucose deprivation/reoxygenation (Qiao et al. 2023), this is the first report demonstrating the anti‐apoptotic effects of liensinine in endothelial cells. Notably, we also observed that this protective effect was accompanied by reduced intracellular ROS levels and increased NO production, suggesting that the endogenous antioxidant system may mediate the vascular protective actions of liensinine.

The Nrf2/HO‐1 pathway is a well‐characterized signaling cascade renowned for its robust anti‐oxidative stress activity, particularly in the context of cardio‐cerebrovascular ischemic diseases. As a main regulator for inhibiting oxidative stress, Nrf2 is combined with Keap1 in an inactive condition. Upon exposure to stress stimuli, Keap1 undergoes post‐translational modifications that trigger Nrf2 activation. Subsequently, Nrf2 translocates to the nucleus, combines with antioxidant response elements, and drives their transcription, thereby exerting cytoprotective effects (Zhou et al. 2025). As a downstream target of the Nrf2 signaling pathway and a key phase II antioxidant enzyme, HO‐1 acts as a critical inducible stress sensor in cells. By catalyzing the degradation of hemoglobin into CO, Fe^2+^ and biliverdin, HO‐1 elicits a cascade of protective biological effects, including upregulating SOD expression to mitigate oxidative damage and inhibit cellular apoptosis (Zhang et al. 2021). Currently, contradictory findings exist regarding Nrf2 expression patterns under oxidative injury. Zhu reported that Caco‐2 cells exposed to 550 μM H_2_O_2_ for 24 h exhibited elevated Nrf2 expression (Zhu et al. 2025). In contrast, Zhang demonstrated that both in vitro HUVEC incubation with 800 μM H_2_O_2_ and in vivo carotid intima injury led to reduced Nrf2 expression (Zhang, Yang, et al. 2024). Similarly, Yao et al. (2024) and He et al. (2024) observed decreased Nrf2 expression in HUVECs following 24 h of exposure to 100 μM and 1.0 mM H_2_O_2_, respectively. Hada further revealed that nuclear and total Nrf2 levels in HUVECs were closely correlated with the duration of H_2_O_2_‐induced injury (Hada et al. 2020). Specifically, Nrf2 levels increased significantly at 1 h post‐H_2_O_2_ incubation but declined gradually thereafter. In the present study, both nuclear and total Nrf2 levels were reduced after 24 h of oxidative injury, which was consistent with the aforementioned reports. Moreover, the expression of HO‐1, phosphorylated eNOS (p‐eNOS), and SOD2 was similarly downregulated following H_2_O_2_ exposure, whereas liensinine treatment markedly reversed these reductions.

To further verify whether the anti‐oxidative stress effects of liensinine were mediated via the Nrf2/HO‐1 pathway and eNOS, we conducted combination experiments using an NO donor (SNP), an eNOS inhibitor (L‐NAME), and an Nrf2 inhibitor (ML385). Moreover, the EC_50_ of liensinine, inducing the proliferation of HUVECs, was 906 nM (approx. 1 μM) in the present study. However, EC_50_ is the median effective concentration, not the best effective concentration. The results of the EC_50_ experiment showed that 5.0 μM, even 100 μM, could induce the proliferation of both normal cultured and H_2_O_2_‐injured cells. Moreover, the results of cellular migration, apoptosis, generations of ROS and NO indicated that 5.0 μM of liensinine possessed better protection than 1.0 μM. So, we chose 5.0 μM of liensinine for the research of Nrf2/HO‐1 signaling and eNOS regulations. The results showed that SNP did not further enhance the protective effects of liensinine, while L‐NAME and ML385 significantly abrogated these effects. Intriguingly, L‐NAME not only decreased p‐eNOS levels but also suppressed Nrf2 expression. Conversely, ML385 not only downregulated Nrf2 levels but also reduced eNOS phosphorylation. Previous studies have reported the crosstalk between the Nrf2/HO‐1 and eNOS pathways. Wang proposed that eNOS acts upstream of the Nrf2/HO‐1 signaling cascade, with the underlying mechanism involving NO‐mediated S‐nitrosylation of Keap1, which reduces Keap1‐Nrf2 binding affinity and promotes Nrf2 dissociation and nuclear translocation (Wang et al. 2013). In contrast, Xiao found that ML385 could reduce eNOS expression in HUVECs and inferred that Nrf2 was involved in the regulation of eNOS (Xiao et al. 2024). Collectively, the findings of the present study provide further evidence that crosstalk may exist between the Nrf2/HO‐1 and eNOS signaling pathways.

Although liensinine has not been used as a commercial supplement currently, more and more studies have elucidated its broad‐spectrum pharmacological activities, including improvement of cardiovascular function (Jia et al. 2023), regulation of energy metabolism (Luo et al. 2025), attenuation of memory impairment (Meng et al. 2022), and mitigation of inflammatory responses (Wang et al. 2024). The present study further confirmed its protection against oxidative stress damage to vascular endothelial cells. To date, no adverse reactions to liensinine have been reported. However, it has been reported to reduce doxorubicin‐induced cardiotoxicity through inhibition of Drp1‐mediated maladaptive mitochondrial fission (Liang et al. 2020). Its multi‐efficacy, low toxicity, and toxicity‐reducing effects suggest its potential as a dietary supplement for many diseases, especially vascular oxidative damage.

## 5 Conclusion

The present study predicted that liensinine, as a main alkaloid from *Plumula nelumbinis*, could ameliorate ODVE by regulating the targets of NFKB1, NFE2L2, ESR1, SRC, MMP9, and CCND1 via bioinformatics analysis. After construction of an H_2_O_2_‐induced ODVE in vitro model, liensinine exhibited an obvious protective effect, including enhancing cellular activities, migration, and NO generation, and reducing ROS production and cellular apoptosis. The potential mechanism might be related to the upregulation of Nrf2/HO‐1 and eNOS signaling, which even possesses cross‐talk modulation. Considering the limitation of the H_2_O_2_—injured model, the other in vitro model (such as oxygen–glucose deprivation/reoxygenation) and in vivo experiments should be performed to further explore the protective effects of liensinine on human vascular endothelial cells.

## Author Contributions

**Feng Gao:** conceptualization, funding acquisition, data curation. **Shuang Liu:** investigation. **Fengjiao Deng:** methodology, funding acquisition. **Yayi Deng:** methodology. **Chenxiao Shan:** visualization, writing – original draft. **Qiaowei Du:** data curation. **Bin Yu:** funding acquisition, writing – review and editing, supervision. **Tianya Xia:** software.

## Funding

This work was supported by the Wuxi Municipal Health Commission Major Scientific Research Project 2024 (Z202306), the National Natural Science Foundation of China (82374316), and the Graduate Research and Innovation Projects of Jiangsu Province (KYCX24_2292).

## Conflicts of Interest

The authors declare no conflicts of interest.

## Data Availability

The data that support the findings of this study are available from the corresponding author upon reasonable request.

## References

[fsn372013-bib-0001] Abrashev, H. , D. Abrasheva , N. Nikolov , J. Ananiev , and E. Georgieva . 2025. “A Systematic Review of Endothelial Dysfunction in Chronic Venous Disease‐Inflammation, Oxidative Stress, and Shear Stress.” International Journal of Molecular Sciences 26, no. 8: 3660. 10.3390/ijms26083660. 40332237 PMC12026777

[fsn372013-bib-0002] Bai, Y. , D. Tan , Q. Deng , et al. 2025. “Cinnamic Acid Alleviates Endothelial Dysfunction and Oxidative Stress by Targeting PPARδ in Obesity and Diabetes.” Chinese Medicine 20, no. 1: 13. 10.1186/s13020-025-01064-7. 39856769 PMC11760083

[fsn372013-bib-0003] Baldea, I. , I. Teacoe , D. E. Olteanu , et al. 2018. “Effects of Different Hypoxia Degrees on Endothelial Cell Cultures‐Time Course Study.” Mechanisms of Ageing and Development 172: 45–50. 10.1016/j.mad.2017.11.003. 29155057

[fsn372013-bib-0004] Cao, J. , J. Ling , J. Cui , Y. Zhao , Z. Xu , and Y. Wang . 2025. “Baicalin Alleviates H2O2‐Induced Oxidative Stress Injury in Human Umbilical Vein Endothelial Cells by Regulating the TNF‐α‐Mediated MAPK/ERK1/2/MPO Pathway.” Scientific Reports 15, no. 1: 35177. 10.1038/s41598-025-19105-4. 41062659 PMC12508144

[fsn372013-bib-0005] Chao, T. Y. , Y. L. Chou , P. T. Young , and T. Q. Chou . 1962. “Studies on the Alkaloids of Embryo Loti, Nelumbo Nucifera Gaertn. I. Isolation and Characterisation of Liensinine.” Scientia Sinica 11: 215–219. 13878148

[fsn372013-bib-0006] Chen, D. , P. Jia , M. Wang , et al. 2025. “Liensinine Can Improve Vascular Remodeling in Hypertension Through the Ferroptosis‐Related TLR4 Inflammatory Pathway.” Journal of Molecular Medicine (Berlin) 104, no. 1: 13. 10.1007/s00109-025-02624-y. PMC12743113 41452356

[fsn372013-bib-0007] Chen, G. L. , M. X. Fan , J. L. Wu , N. Li , and M. Q. Guo . 2019. “Antioxidant and Anti‐Inflammatory Properties of Flavonoids From Lotus Plumule.” Food Chemistry 277: 706–712. 10.1016/j.foodchem.2018.11.040. 30502207

[fsn372013-bib-0008] Chen, S. , W. Guo , X. Qi , J. Zhou , Z. Liu , and Y. Cheng . 2019. “Natural Alkaloids From Lotus Plumule Ameliorate Lipopolysaccharide‐Induced Depression‐Like Behavior: Integrating Network Pharmacology and Molecular Mechanism Evaluation.” Food & Function 10, no. 9: 6062–6073. 10.1039/c9fo01092k. 31486445

[fsn372013-bib-0009] Chen, W. Z. , S. J. Ling , and K. S. Ting . 1962. “Hypotensive Action of Liensinine and Its Two Derivatives.” Acta Pharmaceutica Sinica 9: 277–280. 14075895

[fsn372013-bib-0010] Cheng, D. , X. Liu , Y. Gao , et al. 2023. “α‐Ketoglutarate Attenuates Hyperlipidemia‐Induced Endothelial Damage by Activating the Erk‐Nrf2 Signaling Pathway to Inhibit Oxidative Stress and Mitochondrial Dysfunction.” Antioxidants & Redox Signaling 39, no. 10–12: 777–793. 10.1089/ars.2022.0215. 37154729

[fsn372013-bib-0011] Feng, J. , Q. Tao , Z. J. Zhang , Q. F. Yu , Y. J. Yang , and J. Y. Li . 2025. “Aspirin Eugenol Ester Alleviates Vascular Endothelial Ferroptosis by Enhancing Antioxidant Ability and Inhibiting the JNK/c‐Jun/NCOA4/FTH Signaling Pathway.” Antioxidants 14, no. 10: 1220. 10.3390/antiox14101220. 41154529 PMC12561717

[fsn372013-bib-0012] Gao, J. , M. R. Y. Rouzi , H. Zhang , et al. 2024. “Association of Serum CTRP4 Levels With Vascular Endothelial Function in Patients With Type 2 Diabetes Mellitus: CTRP4 Ameliorating Inflammation, Proliferation and Migration in Human Umbilical Vein Endothelial Cells.” Acta Diabetologica 61, no. 5: 565–575. 10.1007/s00592-023-02228-3. 38286878 PMC11055794

[fsn372013-bib-0013] Guan, G. , H. Han , Y. Yang , Y. Jin , X. Wang , and X. Liu . 2014. “Neferine Prevented Hyperglycemia‐Induced Endothelial Cell Apoptosis Through Suppressing ROS/Akt/NF‐κB Signal.” Endocrine 47, no. 3: 764–771. 10.1007/s12020-014-0186-1. 24590293

[fsn372013-bib-0014] Hada, Y. , H. A. Uchida , N. Otaka , et al. 2020. “The Protective Effect of Chlorogenic Acid on Vascular Senescence via the Nrf2/HO‐1 Pathway.” International Journal of Molecular Sciences 21, no. 12: 4527. 10.3390/ijms21124527. 32630570 PMC7350250

[fsn372013-bib-0015] He, Q. , Y. P. Chen , C. Wu , et al. 2025. “Aloin Protects Against UVB‐Induced Apoptosis by Modulating Integrated Signaling Pathways.” Frontiers in Pharmacology 16: 1584233. 10.3389/fphar.2025.1584233. 40717965 PMC12289626

[fsn372013-bib-0016] He, X. , M. Wu , L. Chen , et al. 2024. “APMCG‐1 Attenuates Ischemic Stroke Injury by Reducing Oxidative Stress and Apoptosis and Promoting Angiogenesis via Activating PI3K/AKT Pathway.” Biomedical Pharmacotherapy 180: 117506. 10.1016/j.biopha.2024.117506. 39368213

[fsn372013-bib-0017] Incalza, M. A. , R. D'Oria , A. Natalicchio , S. Perrini , L. Laviola , and F. Giorgino . 2018. “Oxidative Stress and Reactive Oxygen Species in Endothelial Dysfunction Associated With Cardiovascular and Metabolic Diseases.” Vascular Pharmacology 100: 1–19. 10.1016/j.vph.2017.05.005. 28579545

[fsn372013-bib-0018] Jia, P. , D. Chen , Y. Zhu , et al. 2023. “Liensinine Improves AngII‐Induced Vascular Remodeling via MAPK/TGF‐β1/Smad2/3 Signaling.” Journal of Ethnopharmacology 317: 116768. 10.1016/j.jep.2023.116768. 37308031

[fsn372013-bib-0019] Li, B. , Y. Wu , S. Liao , et al. 2026. “Machine Learning Integrates Bulk and Single‐Nucleus RNA Sequence to Explore Apoptosis‐Related Gene in Myocardial Infarction.” Cardiovascular Therapeautic 1: e5553167. 10.1155/cdr/5553167. PMC13140870 41852026

[fsn372013-bib-0020] Li, S. , B. Zhang , and N. Zhang . 2011. “Network Target for Screening Synergistic Drug Combinations With Application to Traditional Chinese Medicine.” BMC Systems Biology 5, no. Suppl 1: S10. 10.1186/1752-0509-5-S1-S10. PMC3121110 21689469

[fsn372013-bib-0021] Liang, X. , S. Wang , L. Wang , A. F. Ceylan , J. Ren , and Y. Zhang . 2020. “Mitophagy Inhibitor Liensinine Suppresses Doxorubicin‐Induced Cardiotoxicity Through Inhibition of Drp1‐Mediated Maladaptive Mitochondrial Fission.” Pharmacological Research 157: 104846. 10.1016/j.phrs.2020.104846. 32339784

[fsn372013-bib-0022] Liu, B. , J. Li , R. Yi , J. Mu , X. Zhou , and X. Zhao . 2019. “Preventive Effect of Alkaloids From Lotus Plumule on Acute Liver Injury in Mice.” Food 8, no. 1: 36. 10.3390/foods8010036. PMC6352077 30669459

[fsn372013-bib-0023] Luo, Y. , X. Liu , Y. Zhang , et al. 2025. “Liensinine Alleviates Type 2 Diabetes Mellitus Through Modulating the Pancreatic β Cell Function and Gut Microbiota.” Genes & Nutrition 20, no. 1: 29. 10.1186/s12263-025-00789-2. 41444518 PMC12729215

[fsn372013-bib-0024] Ma, S. , Y. Ge , Z. Xiong , et al. 2023. “A Novel Gene Signature Related to Oxidative Stress Predicts the Prognosis in Clear Cell Renal Cell Carcinoma.” PeerJ 11: e14784. 10.7717/peerj.14784. 36785707 PMC9921988

[fsn372013-bib-0025] Meng, X. L. , S. Y. Liu , J. S. Xue , et al. 2022. “Protective Effects of Liensinine, Isoliensinine, and Neferine on PC12 Cells Injured by Amyloid‐β.” Journal of Food Biochemistry 46, no. 10: e14303. 10.1111/jfbc.14303. 35762411

[fsn372013-bib-0026] Michaeli, D. T. , J. C. Michaeli , S. Albers , T. Boch , and T. Michaeli . 2023. “Established and Emerging Lipid‐Lowering Drugs for Primary and Secondary Cardiovascular Prevention.” American Journal of Cardiovascular Drugs 23, no. 5: 477–495. 10.1007/s40256-023-00594-5. 37486464 PMC10462544

[fsn372013-bib-0027] Passacquale, G. , P. Sharma , D. Perera , and A. Ferro . 2022. “Antiplatelet Therapy in Cardiovascular Disease: Current Status and Future Directions.” British Journal of Clinical Pharmacology 88, no. 6: 2686–2699. 10.1111/bcp.15221. 35001413 PMC9303765

[fsn372013-bib-0028] Qiao, W. , Z. Zang , D. Li , S. Shao , Q. Li , and Z. Liu . 2023. “Liensinine Ameliorates Ischemia‐Reperfusion‐Induced Brain Injury by Inhibiting Autophagy via PI3K/AKT Signaling.” Functional & Integrative Genomics 23, no. 2: 140. 10.1007/s10142-023-01063-7. 37118322

[fsn372013-bib-0029] Ruan, M. , G. Lv , X. Wang , et al. 2024. “Network Pharmacology and Validation of the Combinative Therapy of Ligusticum Striatum DC. And Borneolum Against Cerebral Ischemia.” Combinatorial Chemistry & High Throughput Screening 28: 2652–2666. 10.2174/0113862073317255240902075511. 39301903

[fsn372013-bib-0030] Shen, F. , C. Wu , X. Zhong , et al. 2023. “Liensinine Prevents Ischemic Injury Following Myocardial Infarction via Inhibition of Wnt/β‐Catenin Signaling Activation.” Biomedicine & Pharmacotherapy 162: 114675. 10.1016/j.biopha.2023.114675. 37044026

[fsn372013-bib-0031] Tang, D. , M. Chen , X. Huang , et al. 2023. “SRplot: A Free Online Platform for Data Visualization and Graphing.” PLoS One 18, no. 11: e0294236. 10.1371/journal.pone.0294236. 37943830 PMC10635526

[fsn372013-bib-0032] Tang, Y. S. , Y. H. Zhao , Y. Zhong , et al. 2019. “Neferine Inhibits LPS‐ATP‐Induced Endothelial Cell Pyroptosis via Regulation of ROS/NLRP3/Caspase‐1 Signaling Pathway.” Inflammation Research 68, no. 9: 727–738. 10.1007/s00011-019-01256-6. 31172209

[fsn372013-bib-0033] Thannickal, V. J. , and B. L. Fanburg . 2000. “Reactive Oxygen Species in Cell Signaling.” American Journal of Physiology. Lung Cellular and Molecular Physiology 279, no. 6: L1005–L1028. 10.1152/ajplung.2000.279.6.L1005. 11076791

[fsn372013-bib-0034] Uruski, P. , J. Mikuła‐Pietrasik , A. Tykarski , and K. Książek . 2025. “The TGF‐β1‐Oxidative Stress Axis Underlies Accelerated Senescence of Endothelial Cells Exposed to Serum From Hypertensive Patients.” Mechanisms of Ageing and Development 229: 112128. 10.1016/j.mad.2025.112128. 41314306

[fsn372013-bib-0035] Wang, G. , Y. Sun , Q. Yang , et al. 2023. “Liensinine, a Alkaloid From Lotus Plumule, Mitigates Lipopolysaccharide‐Induced Sepsis‐Associated Encephalopathy Through Modulation of Nuclear Factor Erythroid 2‐Related Factor‐Mediated Inflammatory Biomarkers and Mitochondria Apoptosis.” Food and Chemical Toxicology 177: 113813. 10.1016/j.fct.2023.113813. 37150347

[fsn372013-bib-0036] Wang, H. , Y. Yang , X. Zhang , et al. 2023. “Liensinine Attenuates Inflammation and Oxidative Stress in Spleen Tissue in an LPS‐Induced Mouse Sepsis Model.” Journal of Zhejiang University. Science. B 24, no. 2: 185–190. 10.1631/jzus.B2200340. 36751703 PMC10260283

[fsn372013-bib-0037] Wang, L. , T. Shao , C. Liu , et al. 2024. “Liensinine Inhibits IL‐1β‐Stimulated Inflammatory Response in Chondrocytes and Attenuates Papain‐Induced Osteoarthritis in Rats.” International Immunopharmacology 138: 112601. 10.1016/j.intimp.2024.112601. 38971106

[fsn372013-bib-0038] Wang, R. , J. Tu , Q. Zhang , et al. 2013. “Genistein Attenuates Ischemic Oxidative Damage and Behavioral Deficits via eNOS/Nrf2/HO‐1 Signaling.” Hippocampus 23, no. 7: 634–647. 10.1002/hipo.22126. 23536494

[fsn372013-bib-0039] Wang, X. , X. Song , H. Ding Z , et al. 2025. “Jun Ginger Extract Improves Cold‐Induced Asthma by Inhibiting Airway Inflammation via PI3K/AKT Pathway.” Food Science & Nutrition 13, no. 11: e71249. 10.1002/fsn3.71249. 41280738 PMC12629912

[fsn372013-bib-0040] Welsh, P. , G. Grassia , S. Botha , N. Sattar , and P. Maffia . 2017. “Targeting Inflammation to Reduce Cardiovascular Disease Risk: A Realistic Clinical Prospect?” British Journal of Pharmacology 174, no. 22: 3898–3913. 10.1111/bph.13818. 28409825 PMC5660005

[fsn372013-bib-0041] Xiao, F. , S. Rui , X. Zhang , et al. 2024. “Accelerating Diabetic Wound Healing With Ramulus Mori (Sangzhi) Alkaloids via NRF2/HO‐1/eNOS Pathway.” Phytomedicine 134: 155990. 10.1016/j.phymed.2024.155990. 39243750

[fsn372013-bib-0042] Yao, Z. , K. Xue , J. Chen , et al. 2024. “Biliverdin Improved Angiogenesis and Suppressed Apoptosis via PI3K/Akt‐Mediated Nrf2 Antioxidant System to Promote Ischemic Flap Survival.” Free Radical Biology and Medicine 225: 35–52. 10.1016/j.freeradbiomed.2024.09.042. 39332540

[fsn372013-bib-0043] Zhang, Q. , J. Liu , H. Duan , R. Li , W. Peng , and C. Wu . 2021. “Activation of Nrf2/HO‐1 Signaling: An Important Molecular Mechanism of Herbal Medicine in the Treatment of Atherosclerosis via the Protection of Vascular Endothelial Cells From Oxidative Stress.” Journal of Advanced Research 34: 43–63. 10.1016/j.jare.2021.06.023. 35024180 PMC8655139

[fsn372013-bib-0044] Zhang, W. , T. Wang , H. Chen , et al. 2023. “Liensinine Alleviates Septic Heart Injury by Targeting Inflammation, Oxidative Stress, Apoptosis, and Autophagy.” Acta Biochimica et Biophysica Sinica 55, no. 3: 521–524. 10.3724/abbs.2023044. 36951482 PMC10160220

[fsn372013-bib-0045] Zhang, X. , M. Chen , R. Wang , et al. 2026. “Flavonoids: Potential New Drug Candidates for Attenuating Vascular Remodeling in Pulmonary Hypertension.” International Journal of Molecular Sciences 27, no. 1: 210. 10.3390/ijms27010210. PMC12785443 41516089

[fsn372013-bib-0046] Zhang, X. , J. Yang , Y. Lu , Y. Liu , T. Wang , and F. Yu . 2024. “Human Urinary Kallidinogenase Improves Vascular Endothelial Injury by Activating the Nrf2/HO‐1 Signaling Pathway.” Chemico‐Biological Interactions 403: 111230. 10.1016/j.cbi.2024.111230. 39244186

[fsn372013-bib-0047] Zhang, X. , S. Yuan , H. Fan , W. Zhang , and H. Zhang . 2024. “Liensinine Alleviates Sepsis‐Induced Acute Liver Injury by Inhibiting the NF‐κB and MAPK Pathways in an Nrf2‐Dependent Manner.” Chemico‐Biological Interactions 396: 111030. 10.1016/j.cbi.2024.111030. 38692452

[fsn372013-bib-0048] Zhao, H. , M. Miller , K. Pfeiffer , J. A. Buras , and G. L. Stahl . 2003. “Anoxia and Reoxygenation of Human Endothelial Cells Decrease Ceramide Glucosyltransferase Expression and Activates Caspases.” FASEB Journal 17, no. 6: 723–724. 10.1096/fj.02-0806fje. 12586734

[fsn372013-bib-0049] Zhong, K. Y. , Y. Zhang , N. D. Wang , G. W. Li , X. J. Zhang , and Z. J. Yang . 2025. “Effects and Mechanisms of Dietary Natural Products on Ischemic Stroke: An Updated Review.” Food Science & Nutrition 13, no. 12: e71324. 10.1002/fsn3.71324. 41394546 PMC12696334

[fsn372013-bib-0050] Zhou, H. M. , Y. Chai , X. Mao , et al. 2025. “Regression of Oxidative Stress by Targeting Nrf2/HO‐1 Signaling: The Potential Therapeutic Drugs for Cerebral Ischemia‐Reperfusion Injury.” Biomedicine & Pharmacotherapy 193: 118809. 10.1016/j.biopha.2025.118809. 41275622

[fsn372013-bib-0051] Zhou, Y. , S. Tai , N. Zhang , L. Fu , and Y. Wang . 2023. “Dapagliflozin Prevents Oxidative Stress‐Induced Endothelial Dysfunction via Sirtuin 1 Activation.” Biomedicine & Pharmacotherapy 165: 115213. 10.1016/j.biopha.2023.115213. 37517289

[fsn372013-bib-0052] Zhu, Y. Y. , M. M. H. Wu , Z. C. Zhao , et al. 2025. “An Antioxidative Exopolysaccharide‐Protein Complex of Cordyceps cs‐HK1 Fungus and Its Epithelial Barrier‐Protective Effects in Caco‐2 Cell Culture.” Antioxidants (Basel) 14, no. 12: 1501. 10.3390/antiox14121501. 41462700 PMC12729385

